# Associations Between Blood Metal Exposure and Hypertriglyceridemia Among Adults in NHANES, 2011–2018

**DOI:** 10.1002/fsn3.71001

**Published:** 2025-09-21

**Authors:** Xue Zhao, Bin Liu, Hongqi He, Linwei Zhou, Xinjie Gong, Yuhan Fan, Xin Xu, Xu Chai, Shuli An, Xia Chu

**Affiliations:** ^1^ Department of Nutrition and Food Hygiene, School of Public Health Harbin Medical University Heilongjiang China; ^2^ Key Laboratory of Precision Nutrition and Health, Ministry of Education, School of Public Health Harbin Medical University Heilongjiang China; ^3^ National Health Commission Specialty Laboratory Cooperation Unit of Food Safety Risk Assessment and Standard Development, School of Public Health Harbin Medical University Heilongjiang China; ^4^ Heilongjiang Academy of Medical Sciences Harbin China

**Keywords:** co‐exposure, combined effects, hypertriglyceridemia, metal, triglyceride

## Abstract

Growing evidence indicates associations between blood metal exposure and dyslipidemia. However, the relationships between hypertriglyceridemia (HTG), the most prevalent form of dyslipidemia, and blood metal exposure remain unclear. Our study aimed to evaluate both single and combined effects of blood metals on HTG risk. This study included 4182 adults from the 2011–2018 National Health and Nutrition Examination Survey. We assessed single and combined associations between blood metals (cadmium, mercury, lead, selenium, and manganese) and HTG. In single metal analysis, elevated blood selenium concentrations were significantly associated with increased HTG risk and triglyceride (TG) levels, with OR (95% CI) and β (95% CI) of 1.78 (1.36, 2.32) and 0.21 (0.13, 0.29), respectively, in the highest tertile group. Restricted cubic splines revealed a positive linear correlation between selenium and HTG. No significant associations were observed between HTG and blood cadmium, mercury, lead, or manganese in the overall population. In metal mixture analysis, blood metal co‐exposure was positively related to HTG risk across WQS, qgcomp, and BKMR models, particularly in females and individuals under 60 years old. Furthermore, selenium was identified as the primary contributor to the overall mixture effect. The positive directional influence of selenium was further confirmed on the overall effect. Collectively, these findings indicate that elevated blood selenium concentrations are associated with increased HTG risk and higher TG levels. The results provide novel epidemiological evidence for the associations between multi‐metal co‐exposure and HTG risk, particularly in females and individuals under 60 years old.

## Graphical abstract

Elevated blood Se concentrations are associated with increased HTG risk and higher TG levels. Our study provides novel epidemiological evidence for the associations between multi‐metal co‐exposure and HTG risk, particularly in females and individuals under 60 years old.

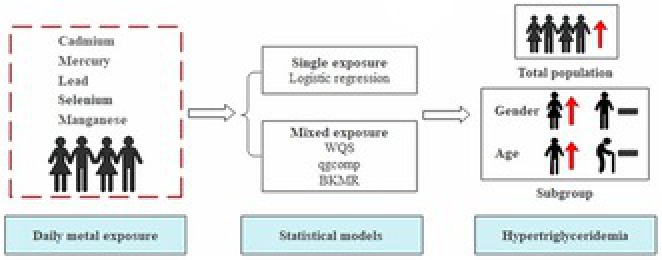

**Abbreviations**
ALT: alanine aminotransferase
AST: aspartate aminotransferase
BKMR: Bayesian kernel machine regression
BMI: body mass index
Cd: cadmium
CI: confidence intervals
CKD: chronic kidney disease
eGFR: estimated Glomerular Filtration Rate
FIPR: family income‐to‐poverty ratio
HDL: high‐density lipoprotein
HEI‐2015: Healthy Eating Index 2015
Hg: mercury
HTG: hypertriglyceridemia
IR: insulin resistance
LDL: low‐density lipoprotein
LOD: limit of detection
LPL: lipoprotein lipase
Mn: manganese
NHANES: National Health and Nutrition Examination Survey
OR: odds ratio
Pb: lead
qgcomp: quantile‐based g‐computation
RCS: restricted cubic splines
ROS: reactive oxygen species
SCFA: short‐chain fatty acid
SD: standard deviation
Se: selenium
TG: triglyceride
VIF: variance inflation factor
WQS: weighted quantile sum

## 1 Introduction

Approximately 40%–50% of American adults have dyslipidemia (Saydah et al. 2014). Hypertriglyceridemia (HTG) represents the most prevalent form of dyslipidemia among adults (Simha 2020). As a significant global public health concern, HTG affects 25%–50% of the worldwide population (Luna‐Castillo et al. 2022). Severe HTG is related to a 33%–38% increase in annual healthcare expenditures, imposing a substantial economic burden on healthcare systems (Nichols et al. 2011). Furthermore, HTG is strongly associated with increased atherogenic remnant lipoprotein particles, nonalcoholic fatty liver disease progression, and elevated pancreatitis risk (Luna‐Castillo et al. 2022). Therefore, identifying modifiable risk factors for triglyceride (TG) dysmetabolism is crucial for preventing HTG onset and progression.

Metal pollution has risen to prominence as a pressing population health challenge, with metals from both anthropogenic and natural sources being pervasive in the environment. These metals enter the human body through multiple pathways, including food, water, and air, and subsequently accumulate in various tissues (Tchounwou et al. 2012). Emerging evidence suggests significant relationships between metal exposure and dysregulated TG metabolism. For example, cadmium (Cd) exposure has been linked to elevated circulating TG levels (Kim et al. 2018). Increased blood lead (Pb) levels show significant positive correlations with TG levels (Park et al. 2019). Notably, epidemiological studies have reported conflicting associations between selenium (Se) and TG levels. A cross‐sectional study has revealed a positive Se‐TG correlation, and Huang et al. have found higher odds of elevated TG with increased Se levels (Bleys et al. 2008; Huang et al. 2020). However, clinical trials in hemodialysis patients and a Danish cohort study have found no association between Se and TG levels (Atapour et al. 2022; Suadicani et al. 1992). Current studies have primarily focused on single‐metal effects on TG metabolism, overlooking potential synergistic or antagonistic effects in metal mixtures. Moreover, the associations between individual and multi‐metal exposures and HTG remain unexplored. Therefore, further investigation is warranted to elucidate the complex interplay between metal exposure and HTG risk.

Absorbed metals undergo systemic distribution through the circulatory system. Blood metal concentrations directly reflect recent exposure status and current circulating metal levels. In this study, we utilized data from the National Health and Nutrition Examination Survey (NHANES) to investigate the associations between HTG and blood concentrations of five metals—Cd, mercury (Hg), Pb, Se, and manganese (Mn)—both individually and as mixtures. We simultaneously employed weighted quantile sum (WQS) regression, quantile‐based g‐computation (qgcomp), and Bayesian kernel machine regression (BKMR) models to assess joint effects of metal mixtures on HTG risk. Additionally, our study evaluated the effects of multi‐metal exposure on HTG within a representative population, with particular focus on potential variations across age and gender strata, to provide deeper insights into the health effects of metal exposure.

## 2 Methods

### 2.1 Study Design and Participants

The NHANES is a representative study conducted to evaluate health‐related data in the United States. Detailed methodologies for sampling and data collection have been provided elsewhere (CDC 2006). This study was approved by the Institutional Review Board at the National Center for Health Statistics. Before their participation, all individuals provided written informed consent.

**FIGURE 1:**
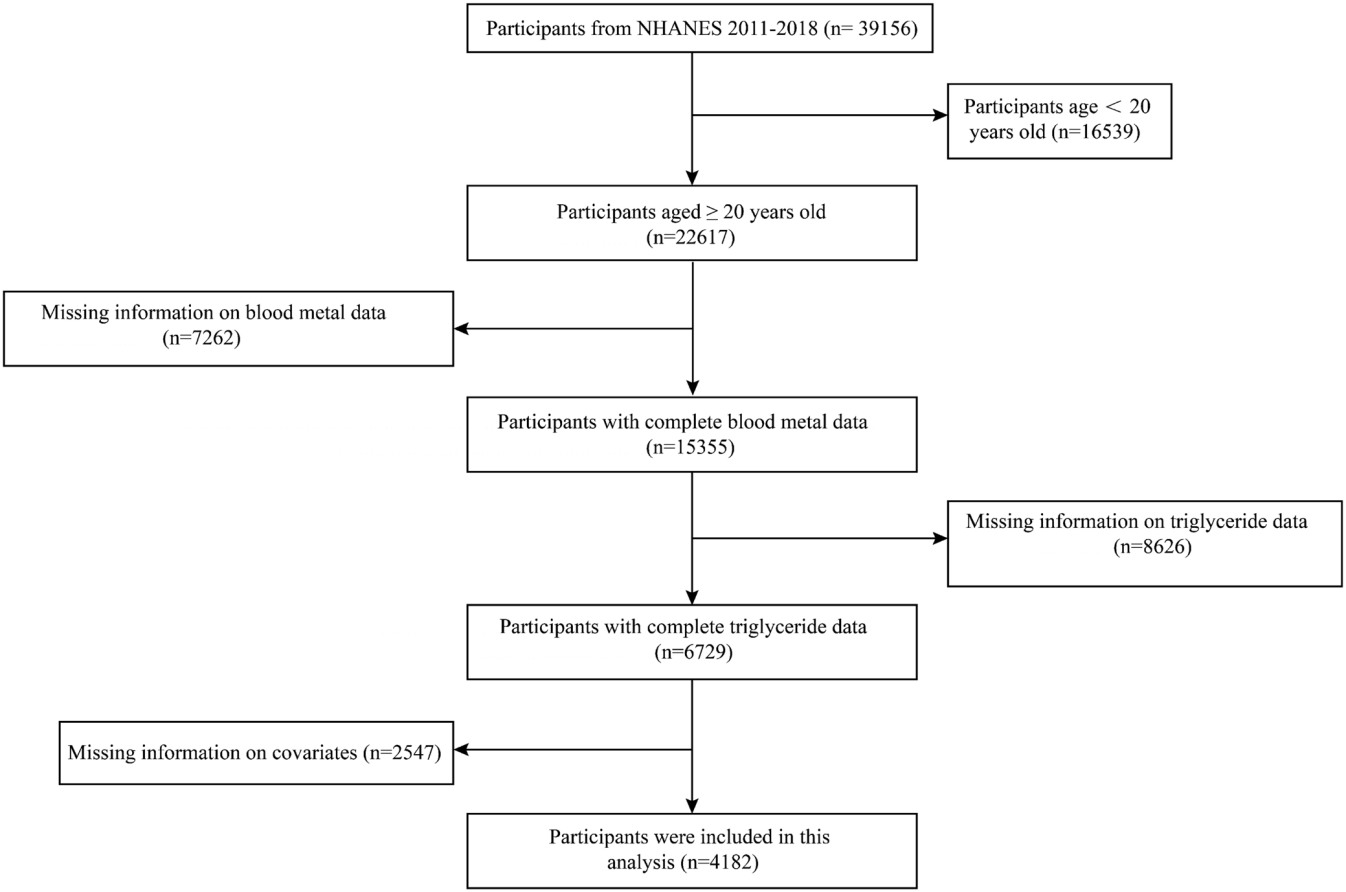
The flowchart of analytic sample selection.

We utilized data from four 2‐year cycles of NHANES conducted between 2011 and 2018. After excluding participants under 20 years of age and individuals with missing information on blood metal data, TG, and covariates, our statistical analysis included 4182 participants with complete measurements of blood metal and TG (Figure 1).

### 2.2 Assessment of Hypertriglyceridemia

TG is measured by the Beckman Synchron LX20 using a timed endpoint method. HTG is defined as TG levels of ≥ 150 mg/dL (Hegele et al. 2014).

### 2.3 Measurement of Blood Metal

Researchers measured blood Pb, Cd, Hg, Se, and Mn concentrations using quadrupole ICP‐MS technology. Blood samples were introduced into the ICP‐MS system via pneumatic atomization using an argon carrier gas. An argon plasma, with a temperature ranging from 6000 to 8000 K and generated through radio‐frequency coupling, evaporated, atomized, and ionized the droplets. The ions were transferred through a differential pressure interface (from 760 Torr to 10^−5^ Torr), then passed through the focusing region, dynamic reaction cell, and quadrupole mass filter before being quantified by a pulse‐counting detector. In addition to the collected samples, blanks, calibration standards, and quality control samples were analyzed. The internal standards (rhodium, iridium, tellurium) were corrected for instrumental noise and drift via metal/internal standard signal ratio. Quality control limits were established from ≥ 20 independent analytical runs. The detection frequency of all exposures was above 85% in Table S1, and values below the limit of detection (LOD) were calculated as the lowest LOD divided by the square root of two. More details can be found in the previous studies (Chen et al. 2022).

### 2.4 Assessment of Covariates

Our study included the following covariates: age (years), gender (female, male), race/ethnicity (Mexican American, non‐Hispanic White, non‐Hispanic Black, other race), educational level (lower than high school, high school, greater than high school), marital status (married/living with a partner, widowed/divorced/separated, never married), family income‐to‐poverty ratio (FIPR) (< 1.30, 1.30–3.5, ≥ 3.5), body mass index (BMI) (< 25 kg/m^2^, 25–30 kg/m^2^, ≥ 30 kg/m^2^), smoking status (no, yes), drinking alcohol status (no, yes), physical activity (inactive, moderate, vigorous), total energy intake (kcal), Healthy Eating Index 2015 (HEI‐2015), chronic kidney disease (CKD) (no, yes), diabetes (no, yes), and hypertension (no, yes). Smoking status was grouped into smoker (someone who had smoked ≥ 100 cigarettes in their lifetime) or non‐smoker (someone who had smoked < 100 cigarettes in their lifetime). Participants were classified as drinkers if they had consumed ≥ 12 alcoholic drinks in the past year; all others were classified as non‐drinkers. Hypertension was defined by a systolic pressure ≥ 140 mmHg, a diastolic pressure ≥ 90 mmHg, a physician's diagnosis of hypertension, or use of anti‐hypertension medication. Diabetes was identified through a physician's diagnosis of the disease or the use of antidiabetic medication. CKD was defined as an estimated Glomerular Filtration Rate (eGFR) of less than 60 mL/min/1.73 m^2^ (Webster et al. 2017), calculated using the CKD Epidemiology Collaboration formula (Levey et al. 2009). The variance inflation factor (VIF) analysis was conducted to detect multicollinearity among variables. Multicollinearity was not present as VIF remained below the threshold value of 5 in Table S2.

### 2.5 Statistical Analysis

To compare baseline conditions, participants were grouped according to HTG or non‐HTG status. We expressed binary or categorical variables as numbers (%) and continuous variables using the mean and standard deviation (SD). Continuous variables and categorical variables among individuals with and without HTG were tested using the Wilcoxon test and Rao‐Scott chi‐square test. The values of the studied metal measurements were ln‐transformed before analysis. Pearson correlation analysis was employed to ascertain the correlations among the five types of blood metal concentrations. Binary logistic regression models and multivariable linear models were utilized to calculate odds ratios (OR), beta estimates, and their corresponding 95% confidence intervals (CI) to explore the relationships between single blood metals and HTG as well as TG, respectively. We used restricted cubic splines (RCS) functions with five nodes (5th, 28th, 50th, 73rd, and 95th percentiles) for dose–response analysis in the fully adjusted model. Subgroup analyses were stratified by age (< 60/≥ 60 years), gender (male/female), and BMI (< 25/25–30/≥ 30 kg/m^2^) to evaluate differences between different groups.

To evaluate joint effects of exposure to five metals on HTG, we employed WQS (Carrico et al. 2015), qgcomp (Keil et al. 2020), and BKMR (Bobb et al. 2015) models. Firstly, a weighted linear index in the WQS model was calculated to evaluate combined effects of metal mixtures. The data were randomly partitioned into training and validation subsets at a 2:3 ratio, followed by 5000 bootstrap iterations to estimate the effect weights of individual metals. The OR from the WQS regression represents the effect of a one‐quartile increment in metal mixtures on HTG risk. Given that WQS regression assumes unidirectional effects of metal exposure and cannot simultaneously evaluate mixtures containing components with positive and negative effect directions, we further implemented the qgcomp model to explore mixture‐outcome relationships. The qgcomp approach eliminates the directional assumption, enabling the estimation of both positive and negative weights for each metal within the mixture. In addition, the BKMR model was employed to evaluate combined effects of metal mixtures by comparing HTG risks when all metals were simultaneously adjusted to different exposure quantiles relative to their median levels. The estimation of model parameters was conducted employing the Markov Chain Monte Carlo approach with 10,000 iterations.

To verify the robustness of our results, we conducted five sensitivity analyses. Firstly, considering that diabetes and hypertension are closely related to abnormal TG metabolism, after excluding participants with diabetes or hypertension, the data were re‐analyzed. In addition, additional adjustments were made for blood Cd, Hg, Pb, Se, and Mn levels to account for interaction among metals. Furthermore, we also performed the above analysis with additional adjustments for dietary iron, dietary magnesium, and drinking water intake to eliminate the effect of diet on TG levels. Finally, analyses were conducted again with additional adjustments for aspartate aminotransferase (AST), alanine aminotransferase (ALT), high‐density lipoprotein (HDL), and low‐density lipoprotein (LDL).

All analyses were performed using R (version 4.4.0). Statistical analyses were two‐sided, and a P value of < 0.05 was considered statistically significant.

## 3 Results

### 3.1 Baseline Characteristics of all Participants

This study included 4182 participants from the 2011–2018 NHANES, comprising 879 HTG cases and 3303 non‐HTG controls. Table 1 presented the baseline characteristics of participants, with significant differences observed between HTG and non‐HTG groups, including gender, race/ethnicity, education level, marital status, BMI, smoking status, total energy intake, physical activity, CKD, diabetes, and hypertension. The interrelationships among the five blood metal concentrations were visually presented in Figure S1. The correlation analysis demonstrated generally weak associations among the different metal concentrations.

**TABLE 1:** Characteristics of participants included in the NHANES 2011–2018 cycles.

Characteristic	Overall (*N* = 4182)	Non‐HTG (*N* = 3303)	HTG (*N* = 879)	*p*
Age (years)	47.74 (16.85)	47.00 (17.28)	50.45 (14.86)	< 0.001
Gender, N (%)				< 0.001
Female	2150 (51.41%)	1771 (53.62%)	379 (43.12%)	
Male	2032 (48.59%)	1532 (46.38%)	500 (56.88%)	
Race/ethnicity				< 0.001
Mexican American	531 (12.70%)	390 (11.81%)	141 (16.04%)	
Non‐Hispanic White	1725 (41.25%)	1321 (39.99%)	404 (45.96%)	
Non‐Hispanic Black	889 (21.26%)	808 (24.46%)	81 (9.22%)	
Other Race	1037 (24.80%)	784 (23.74%)	253 (28.78%)	
Educational level				0.016
Lower than high school	789 (18.87%)	607 (18.38%)	182 (20.71%)	
High school	939 (22.45%)	714 (21.62%)	225 (25.60%)	
Greater than high school	2454 (58.68%)	1982 (60.01%)	472 (53.70%)	
Marital status				< 0.001
Married/living with partner	2515 (60.14%)	1948 (58.98%)	567 (64.51%)	
Never married	873 (20.88%)	671 (20.31%)	202 (22.98%)	
Widowed/divorced/separated	794 (18.99%)	684 (20.71%)	110 (12.51%)	
FIPR				0.9
< 1.30	1270 (30.37%)	993 (30.06%)	277 (31.51%)	
1.30–3.5	1649 (39.43%)	1311 (39.69%)	338 (38.45%)	
> 3.50	1263 (30.20%)	999 (30.25%)	264 (30.03%)	
BMI (kg/m^2^)				< 0.001
< 25	1178 (28.17%)	1045 (31.64%)	133 (15.13%)	
25–30	1364 (32.62%)	1066 (32.27%)	298 (33.90%)	
> 30	1640 (39.22%)	1192 (36.09%)	448 (50.97%)	
Smoking status				0.016
No	2370 (56.67%)	1932 (58.49%)	438 (49.83%)	
Yes	1812 (43.33%)	1371 (41.51%)	441 (50.17%)	
Drinking alcohol status				0.3
No	839 (20.06%)	679 (20.56%)	160 (18.20%)	
Yes	3343 (79.94%)	2624 (79.44%)	719 (81.80%)	
Total energy intake (kcal)	2101.06 (797.94)	2082.77 (792.69)	2167.72 (813.74)	0.028
Physical activity				< 0.001
Inactive	2056 (49.16%)	1556 (47.11%)	500 (56.88%)	
Moderate	1118 (26.73%)	891 (26.98%)	227 (25.82%)	
Vigorous	1008 (24.10%)	856 (25.92%)	152 (17.29%)	
CKD				0.003
No	3504 (83.79%)	2804 (84.89%)	700 (79.64%)	
Yes	678 (16.21%)	499 (15.11%)	179 (20.36%)	
Diabetes				< 0.001
No	3378 (80.77%)	2766 (83.74%)	612 (69.62%)	
Yes	804 (19.23%)	537 (16.26%)	267 (30.38%)	
Hypertension				< 0.001
No	2557 (61.14%)	2095 (63.43%)	462 (52.56%)	
Yes	1625 (38.86%)	1208 (36.57%)	417 (47.44%)	
HEI‐2015	51.35 (12.30)	51.64 (12.52)	50.31 (11.41)	0.064

*Note:* Continuous data were displayed as mean (SD), while categorical variables were exhibited as numbers (percentages).

### 3.2 Associations Between Single Metal Exposure and Triglyceride Levels and Hypertriglyceridemia Risk

Blood metal levels were included in the statistical analysis as both tertile categorical variables and continuous variables. Table 2 presented the relationships between blood Pb, Cd, Hg, Se, Mn, and TG levels. In Model 3, compared to the lowest tertile group (T1), the second (T2) and third (T3) tertile groups of Se had higher TG levels, with β (95% CI) of 0.09 (0.01, 0.17) and 0.21 (0.13, 0.29), respectively. TG levels increased significantly with elevated blood Se concentrations. When Se and Cd were analyzed as continuous variables, β (95% CI) for Se and Cd with TG levels were 0.55 (0.24, 0.86) and 0.05 (0.00, 0.10), respectively.

**TABLE 2:** Associations between blood metal levels and TG in the NHANES 2011–2018 cycles.

Variable	TG β (95% CI)					
	Categorical variable				Continuous variable	
	T1	T2	T3	*p*‐trend	Ln‐transformed	*p*
Pb						
Model 1	Reference	0.09 (0.02, 0.16)	0.09 (0.01, 0.17)	0.022	0.08 (0.03, 0.12)	< 0.001
Model 2	Reference	−0.01 (−0.08, 0.07)	−0.07 (−0.15, 0.02)	0.2	−0.01 (−0.07, 0.04)	0.6
Model 3	Reference	0.01 (−0.06, 0.09)	0 (−0.08, 0.08)	> 0.9	0.04 (−0.02, 0.09)	0.2
Cd						
Model 1	Reference	−0.01 (−0.09, 0.07)	0.05 (−0.03, 0.12)	0.3	0.04 (0.00, 0.07)	0.039
Model 2	Reference	−0.02 (−0.10, 0.06)	0.03 (−0.04, 0.11)	0.4	0.03 (0.00, 0.07)	0.053
Model 3	Reference	0.03 (−0.05, 0.10)	0.06 (−0.04, 0.16)	0.4	0.05 (0.00, 0.10)	0.043
Hg						
Model 1	Reference	0.04 (−0.04, 0.13)	−0.05 (−0.12, 0.02)	0.087	−0.03 (−0.07, 0.00)	0.031
Model 2	Reference	0.02 (−0.07, 0.11)	−0.08 (−0.16, −0.01)	0.013	−0.05 (−0.08, −0.02)	0.001
Model 3	Reference	0.04 (−0.04, 0.12)	0.01 (−0.06, 0.08)	0.6	0 (−0.04, 0.03)	0.8
Se						
Model 1	Reference	0.1 (0.01, 0.19)	0.26 (0.17, 0.34)	< 0.001	0.65 (0.29, 1.0)	< 0.001
Model 2	Reference	0.09 (0.01, 0.18)	0.24 (0.15, 0.32)	< 0.001	0.58 (0.23, 0.92)	< 0.001
Model 3	Reference	0.09 (0.01, 0.17)	0.21 (0.13, 0.29)	< 0.001	0.55 (0.24, 0.86)	< 0.001
Mn						
Model 1	Reference	0 (−0.07, 0.07)	−0.06 (−0.12, 0.00)	0.11	−0.03 (−0.11, 0.04)	0.4
Model 2	Reference	0.04 (−0.03, 0.11)	0.01 (−0.06, 0.07)	0.4	0.06 (−0.02, 0.14)	0.12
Model 3	Reference	0.02 (−0.05, 0.08)	−0.07 (−0.13, −0.01)	0.023	−0.04 (−0.11, 0.04)	0.3

*Note:* Model 1 was a crude model with no adjusted covariates; Model 2 was adjusted for gender and age; Model 3 was adjusted for gender, age, race/ethnicity, FIPR, educational level, smoking status, drinking alcohol status, BMI, physical activity, total energy intake, HEI‐2015, CKD, diabetes, and hypertension.

**FIGURE 2:**
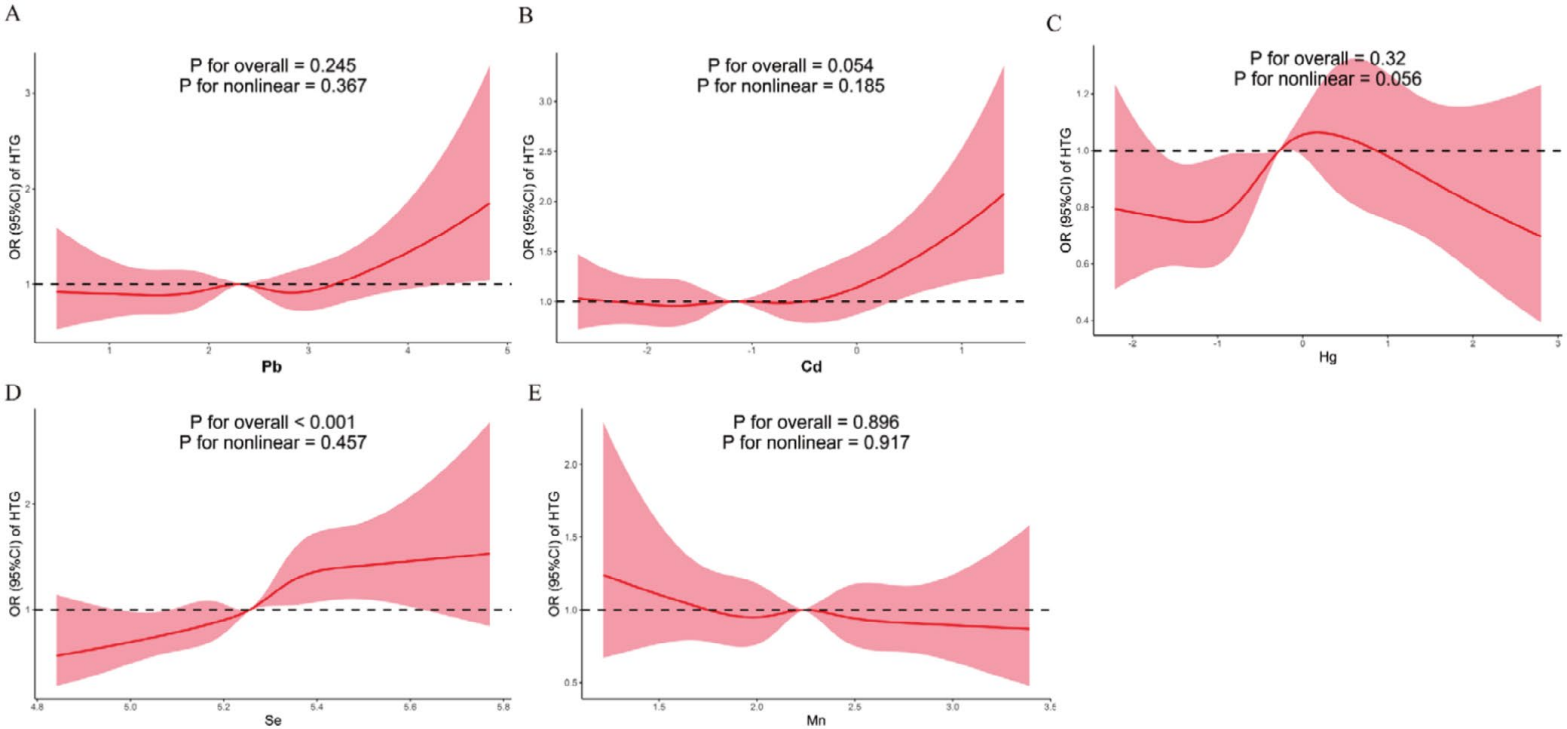
The nonlinear associations between blood metal levels and HTG. The model was adjusted for gender, age, race/ethnicity, FIPR, educational level, smoking status, drinking alcohol status, BMI, physical activity, total energy intake, HEI‐2015, CKD, diabetes, and hypertension.

Table 3 illustrated the correlations between single metals and the risk of HTG. In Model 3, adjusted OR (95% CI) for participants of Se in T3 was 1.78 (1.36, 2.32), compared to T1. When Se concentrations were treated as a continuous variable in the analysis, similar results were observed, with an OR (95% CI) of 4.04 (1.67, 9.80). Moreover, the RCS analysis displayed that blood Se levels were related to the risk of HTG (Figure 2). Higher Se levels showed a significant linear relationship with increased HTG risk (P for overall < 0.001, P for nonlinear = 0.457). However, no relationship between the remaining four metals and HTG was observed.

**TABLE 3:** Associations between blood metal levels and HTG in the NHANES 2011–2018 cycles.

Variable	HTG OR (95% CI)					
	Categorical variable				Continuous variable	
	T1	T2	T3	*p*‐trend	Ln‐transformed	*p*
Pb						
Model 1	Reference	1.14 (0.86, 1.50)	1.08 (0.81, 1.45)	0.7	1.17 (1.00, 1.38)	0.051
Model 2	Reference	0.87 (0.65, 1.16)	0.71 (0.51, 1.0)	0.12	0.92 (0.74, 1.14)	0.4
Model 3	Reference	0.91 (0.67, 1.24)	0.85 (0.61, 1.19)	0.6	1.07 (0.87, 1.31)	0.5
Cd						
Model 1	Reference	1.06 (0.81, 1.39)	1.11 (0.85, 1.44)	0.7	1.05 (0.92, 1.20)	0.4
Model 2	Reference	1.05 (0.79, 1.40)	1.1 (0.83, 1.44)	0.8	1.05 (0.92, 1.20)	0.5
Model 3	Reference	1.22 (0.90, 1.64)	1.28 (0.93, 1.75)	0.2	1.13 (0.96, 1.32)	0.12
Hg						
Model 1	Reference	1.23 (0.97, 1.56)	0.95 (0.74, 1.23)	0.072	0.97 (0.87, 1.08)	0.6
Model 2	Reference	1.19 (0.93, 1.51)	0.88 (0.69, 1.13)	0.052	0.94 (0.84, 1.04)	0.2
Model 3	Reference	1.27 (0.98, 1.65)	1.1 (0.84, 1.45)	0.2	1.05 (0.93, 1.18)	0.4
Se						
Model 1	Reference	1.22 (0.93, 1.61)	1.93 (1.49, 2.50)	< 0.001	4.57 (1.80, 11.6)	0.001
Model 2	Reference	1.19 (0.91, 1.57)	1.84 (1.43, 2.38)	< 0.001	3.84 (1.53, 9.67)	0.004
Model 3	Reference	1.19 (0.89, 1.58)	1.78 (1.36, 2.32)	< 0.001	4.04 (1.67, 9.80)	0.001
Mn						
Model 1	Reference	1.05 (0.84, 1.31)	0.8 (0.66, 0.98)	0.035	0.85 (0.67, 1.09)	0.2
Model 2	Reference	1.16 (0.93, 1.43)	0.94 (0.77, 1.15)	0.2	1.08 (0.84, 1.38)	0.5
Model 3	Reference	1.11 (0.88, 1.40)	0.77 (0.62, 0.95)	0.012	0.84 (0.64, 1.10)	0.2

*Note:* Model 1 was a crude model with no adjusted covariates; Model 2 was adjusted for gender and age; Model 3 was adjusted for gender, age, race/ethnicity, FIPR, educational level, smoking status, drinking alcohol status, BMI, physical activity, total energy intake, HEI‐2015, CKD, diabetes, and hypertension.

### 3.3 Associations Between Co‐Exposure to Multiple Metals and Hypertriglyceridemia Risk

**FIGURE 3:**
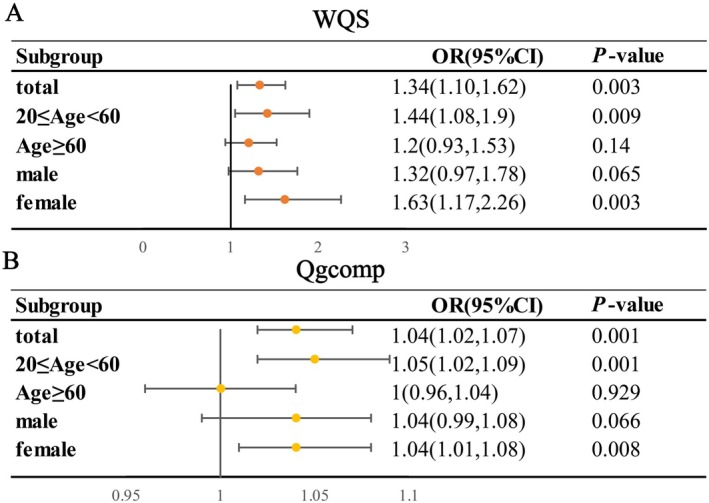
Association of co‐exposure to blood metal levels with HTG in the total population and subgroups by WQS (A) and qgcomp (B) model. The model was adjusted for gender, age, race/ethnicity, FIPR, educational level, smoking status, drinking alcohol status, BMI, physical activity, total energy intake, HEI‐2015, CKD, diabetes, and hypertension.

The WQS analysis (Figure 3A) revealed a significant positive association between a mixture of five metals and HTG risk, with an OR (95% CI) of 1.34 (1.10, 1.62). Stratified analyses demonstrated positive associations in the age < 60 subgroup (1.44 [1.08, 1.90]) and female population (1.63 [1.17, 2.26]). Consistent findings were observed in the qgcomp analysis (Figure 3B), which identified significant associations in the overall population (1.04 [1.02, 1.07]), age < 60 subgroup (1.05 [1.02, 1.09]), and female population (1.04 [1.01, 1.08]). These results were further corroborated by the BKMR model (Figure S2), indicating that exposure to metal mixtures at the 75th percentile was associated with significantly higher HTG risk compared to median exposure levels across the overall population, age < 60 subgroup, and both gender groups.

To quantify single metal contributions, both WQS and qgcomp models were utilized to estimate component weights. The WQS model identified Se as the primary contributor to the overall mixture effect (Figure S3). This observation was further validated by the qgcomp analysis (Figure S4), which confirmed the positive directional influence of Se on the overall effect.

### 3.4 Stratified and Sensitivity Analyses

In subgroup analyses, increased TG levels were observed in the age < 60 group and age ≥ 60 group for Se in T3 compared to T1 (Figure S5), with β (95% CI) of 0.21 (0.11, 0.32) and 0.14 (0.03, 0.26), respectively. Furthermore, statistically significant associations between Se and elevated TG levels were consistently observed in both male and female populations (Figure S6), with β (95% CI) of 0.19 (0.06, 0.32) and 0.19 (0.10, 0.27), respectively. Moreover, positive associations of Se with TG levels remained significant across all BMI strata (Figure S7). In addition, no age‐specific, gender‐specific, or BMI‐specific associations were found between Se and HTG risk (Figures S8–, S10).

In sensitivity analyses, after excluding participants diagnosed with diabetes or hypertension, the remaining sample sizes were 3378 and 2557, respectively, and the results showed no significant changes (Tables S3–, S6). The positive associations between Se and HTG risk were consistently observed, with OR (95% CI) of 1.79 (1.28, 2.52) and 1.72 (1.21, 2.44), respectively. Among the 4182 participants, we observed similar correlations after further adjustment for blood Cd, Hg, Pb, Se, Mn, dietary iron, dietary magnesium, drinking water intake, AST, ALT, HDL, and LDL levels (Tables S7–, S12). The positive associations between Se and HTG risk remained significant, with OR (95% CI) of 1.82 (1.39, 2.37), 1.78 (1.36, 2.32), and 1.52 (1.15, 2.01), respectively.

## 4 Discussion

In this study, we investigated the associations between single and multi‐metal exposures and HTG risk using multiple statistical approaches. Our findings revealed a significant positive correlation between blood Se concentrations and TG levels. Logistic regression analysis identified Se as a potential risk factor for HTG, and RCS modeling further confirmed a linear dose–response relationship between Se exposure and HTG risk. Moreover, consistent results were obtained from WQS, qgcomp, and BKMR models, all indicating positive associations between multi‐metal exposure and HTG risk. Both WQS and qgcomp models highlighted Se as the primary contributor to the overall effect, with qgcomp confirming its significant positive effect. Stratified analyses revealed that the increased HTG risk associated with multi‐metal exposure was more pronounced in females and individuals under 60 years old.

Se, an essential trace element, serves as a critical component of antioxidant enzymes, which play a vital role in neutralizing reactive oxygen species (ROS) and mitigating oxidative stress‐induced cellular damage. However, the biological effects of Se follow a dose‐dependent relationship, with excessive intake posing potential health risks. Epidemiological studies have linked elevated blood Se levels to hypertension (Laclaustra et al. 2009), hyperlipidemia (Ju et al. 2018), hyperglycemia (Cheng et al. 2022), and other metabolic disorders (Li et al. 2024; Wang, Seo, and Park 2021). The correlations between Se and TG levels remain controversial in current epidemiological research. Analysis of NHANES III data revealed a significant positive association between Se concentrations and the prevalence of abnormal TG levels (Bleys et al. 2008). Similarly, Huang et al. demonstrated that individuals with circulating Se levels in the highest quartile had significantly higher odds of elevated TG (Huang et al. 2020). However, a randomized clinical trial in hemodialysis patients found no significant effect of Se supplementation on TG levels (Atapour et al. 2022). A Danish cohort study involving 3387 men with a mean age of 63 years also reported no association between Se and TG levels (Suadicani et al. 1992). In our study, Se showed significant positive associations with both TG levels and HTG risk. Several mechanisms may explain the association between Se and HTG. Firstly, excessive Se intake may induce oxidative stress and generate ROS, leading to lipid peroxidation and elevated TG levels. Additionally, previous research has reported that ROS can induce insulin resistance (IR) (Ayer et al. 2022; Houstis et al. 2006), which is strongly associated with HTG. IR contributes to hepatic TG overproduction and reduces lipoprotein lipase (LPL) activity, thereby impairing TG catabolism. Furthermore, high Se concentrations may activate the NF‐κB inflammatory pathway (Zhang et al. 2019) and promote the release of inflammatory cytokines, such as interleukin‐6 and tumor necrosis factor‐alpha, that indirectly disrupt TG metabolism. In addition, elevated blood Se levels have been linked to increased abundance of Proteobacteria (Shen et al. 2022), a potentially pathogenic bacterial phylum associated with metabolic disorders (Shin et al. 2015). The proliferation of Proteobacteria may suppress the growth of beneficial microbiota responsible for short‐chain fatty acid (SCFA) production. SCFAs, generated through microbial fermentation of dietary fibers in the colon, play crucial roles in lipid homeostasis and may offer protective effects against various lipid metabolism‐associated diseases (Hu et al. 2018; May and den Hartigh 2021). However, further mechanistic studies are needed to fully elucidate the underlying pathways linking Se to HTG.

Our multi‐metal co‐exposure analyses using WQS, qgcomp, and BKMR consistently demonstrated positive associations between metal mixtures and HTG risk. Notably, while the WQS model was constrained in evaluating joint effects with mixed directional associations, the qgcomp approach may result in counteracting effects among metal exposures (Gennings 2021). The combination of these two approaches appeared to reflect the combined effects of multi‐metal exposure more accurately. While only Se was significantly associated with HTG in the overall population, the potential contributions of other metals to HTG required careful consideration in our study. Specifically, our analysis revealed Cd's notable contribution to elevated TG levels, as evidenced by its effect proportion in both WQS and qgcomp models. An interventional study in zebrafish demonstrated that Cd exposure significantly elevated TG levels (Kim et al. 2018). In addition, a Korean study reported that blood Pb levels showed positive associations with TG levels (Park et al. 2019). Metal mixtures may exert complex health effects through additive, synergistic, or antagonistic interactions. Therefore, we hypothesize that certain metals like Cd and Pb, while not primary contributors individually, may influence HTG risk through interactive effects with other metals.

In addition, subgroup analyses revealed that the relationships between multi‐metal exposure and HTG differed by gender and age, being more pronounced in females and individuals under 60 years of age. The precise mechanisms underlying gender‐ and age‐specific differences remain unclear. A previous investigation has demonstrated significant associations between Se and lipid profiles that are particularly influenced by menopausal status in females (Karita et al. 2008). Furthermore, studies have reported notable connections between Pb, Cd, and serum estrogen levels (Gerald et al. 2023; Wang, Ding, et al. 2021). Recent research has identified the crucial biological role of estrogen in regulating TG metabolism (Palmisano et al. 2017). Therefore, we hypothesize that gender‐specific differences may be attributed to variations in sex hormone profiles between genders. The observed age‐related differences may be explained by several factors. Firstly, occupational exposure patterns vary significantly across age subgroups. Younger and middle‐aged individuals are represented in high‐risk occupations, such as metal mining and quarry operations, resulting in higher cumulative toxic metal exposure. Secondly, the young and middle‐aged population tends to exhibit lower health consciousness and higher propensity for unhealthy lifestyles, contributing to increased adiposity (Telleria‐Aramburu and Arroyo‐Izaga 2022). Since metals tend to accumulate in adipose tissue (Attia et al. 2022), impaired metabolism and excretion may further exacerbate their effects. Thirdly, aging is accompanied by increased oxidative stress (Luo et al. 2020). Older individuals exhibit higher baseline oxidative stress levels compared to younger and middle‐aged groups. Metal‐induced oxidative stress is a key factor in HTG development. However, the elevated baseline oxidative stress in older individuals may attenuate the additional effects of metal‐induced oxidative stress, thereby diminishing detectable correlations between metal exposure and HTG risk in the elderly population. Large‐scale prospective studies are warranted to further clarify the associations between multi‐metal exposure and HTG across diverse demographic strata.

Our study provides significant implications for HTG prevention and management in both clinical practice and public health policy. Firstly, in clinical practice, healthcare providers should incorporate assessments of exposure to metal mixtures into the management of HTG patients. The metal‐HTG relationship facilitates early detection and monitoring of HTG and identification of high‐risk individuals. Furthermore, given the ubiquity of co‐exposure to multiple metals in daily life, exposure reduction measures should be implemented. Public health campaigns can be conducted to raise awareness about the exposure risks of metal mixtures and promote lifestyle modifications to mitigate potential health impacts. In addition, although our findings provide important insights into the metal‐HTG relationship, further research through prospective cohort studies and mechanistic experiments is required to establish causal links and elucidate the pathophysiological pathways.

Our study has several benefits. This is the first study to evaluate the associations between both single and multi‐metal exposures and HTG. We employed three statistical methods to assess the effects of multi‐metal exposure on HTG risk. In addition, subgroup analyses revealed specific population susceptibilities to metal exposure effects on HTG risk. Nevertheless, several limitations warrant consideration. Primarily, metals tend to persist in the human body for extended periods due to their long biological half‐lives. Therefore, single measurements may not effectively represent the daily exposure. Secondly, potential confounding factors and measurement inaccuracies may influence our findings. Large‐scale prospective studies should be conducted to validate these observations. Thirdly, the cross‐sectional design limits causal inference, potentially introducing reverse causality bias. Therefore, further longitudinal cohort studies and mechanistic experiments are needed to confirm the causal effects of metal exposure on HTG and elucidate the underlying biological pathways.

## 5 Conclusion

Our findings indicate that elevated blood Se concentrations are associated with increased HTG risk and higher TG levels. The results provide novel epidemiological evidence for associations between multi‐metal co‐exposure and HTG risk, particularly in females and individuals under 60 years old. Longitudinal cohort investigations combined with experimental studies are needed to validate these relationships and elucidate the causal mechanisms.

## Author Contributions

**Xue Zhao:** conceptualization (equal), formal analysis (equal), methodology (equal), writing – original draft (lead), writing – review and editing (equal). **Bin Liu:** conceptualization (equal), formal analysis (equal), methodology (equal), writing – review and editing (equal). **Hongqi He:** methodology (equal), writing – review and editing (equal). **Linwei Zhou:** methodology (equal), writing – review and editing (equal). **Xinjie Gong:** methodology (equal), writing – review and editing (equal). **Yuhan Fan:** methodology (equal), writing – review and editing (equal). **Xin Xu:** methodology (equal), writing – review and editing (equal). **Xu Chai:** methodology (equal), writing – review and editing (equal). **Shuli An:** methodology (equal), writing – review and editing (equal). **Xia Chu:** conceptualization (equal), methodology (equal), supervision (lead), writing – review and editing (equal).

## Ethics Statement

The study complies with the Declaration of Helsinki. The National Center for Health Statistics' Ethics Review Board approved the study (https://www.cdc.gov/nchs/nhanes/about/erb.html?CDC_AAref_Val, accessed on 15 July 2025). Written informed consent is obtained from all participants.

## Conflicts of Interest

The authors declare no conflicts of interest.

## Supporting information


**Figure S1:** The Pearson correlation between blood metals after In‐transformed.

**Figure S2:** Association of co‐exposure to blood metals with hypertriglyceridemia in total population (A) and subgroups (B‐E) by BKMR model. Model was adjusted for gender, age, race/ethnicity, FIPR, educational level, smoking status, drinking alcohol status, BMI, physical activity, total energy intake, HEI‐2015, CKD, diabetes, and hypertension.

**Figure S3:** Estimated weights of blood metals for hypertriglyceridemia in total population (A) and subgroups stratified by age (B‐C) and gender (D‐E) by WQS model. Model was adjusted for gender, age, race/ethnicity, FIPR, educational level, smoking status, drinking alcohol status, BMI, physical activity, total energy intake, HEI‐2015, CKD, diabetes, and hypertension.

**Figure S4:** Estimated weights of blood metals for hypertriglyceridemia in total population (A) and subgroups stratified by age (B‐C) and gender (D‐E) by qgcomp model. Model was adjusted for gender, age, race/ethnicity, FIPR, educational level, smoking status, drinking alcohol status, BMI, physical activity, total energy intake, HEI‐2015, CKD, diabetes, and hypertension.

**Figure S5:** The associations between blood metal levels and triglycerides stratified by age. Model was adjusted for gender, race/ethnicity, FIPR, educational level, smoking status, drinking alcohol status, BMI, physical activity, total energy intake, HEI‐2015, CKD, diabetes, and hypertension.

**Figure S6:** The associations between blood metal levels and triglycerides stratified by gender. Model was adjusted for age, race/ethnicity, FIPR, educational level, smoking status, drinking alcohol status, BMI, physical activity, total energy intake, HEI‐2015, CKD, diabetes, and hypertension.

**Figure S7:** The associations between blood metal levels and triglycerides stratified by BMI. Model was adjusted for gender, age, race/ethnicity, FIPR, educational level, smoking status, drinking alcohol status, physical activity, total energy intake, HEI‐2015, CKD, diabetes, and hypertension.

**Figure S8:** The associations between blood metal levels and hypertriglyceridemia stratified by age. Model was adjusted for gender, race/ethnicity, FIPR, educational level, smoking status, drinking alcohol status, BMI, physical activity, total energy intake, HEI‐2015, CKD, diabetes, and hypertension.

**Figure S9:** The associations between blood metal levels and hypertriglyceridemia stratified by gender. Model was adjusted for age, race/ethnicity, FIPR, educational level, smoking status, drinking alcohol status, BMI, physical activity, total energy intake, HEI‐2015, CKD, diabetes, and hypertension.

**Figure S10:** The associations between blood metal levels and hypertriglyceridemia stratified by BMI. Model was adjusted for gender, age, race/ethnicity, FIPR, educational level, smoking status, drinking alcohol status, physical activity, total energy intake, HEI‐2015, CKD, diabetes, and hypertension.

**Table S1:** The distributions of blood metals in the NHANES 2011–2018.

**Table S2:** Determination of variance inflation factor of the variables.

**Table S3:** Associations between blood metal levels and triglycerides in NHANES excluding the participants with diabetes (*N* = 3378).

**Table S4:** Associations between blood metal levels and hypertriglyceridemia in NHANES excluding the participants with diabetes (*N* = 3378).

**Table S5:** Associations between blood metal levels and triglycerides in NHANES excluding the participants with hypertension (*N* = 2557).

**Table S6:** Associations between blood metal levels and hypertriglyceridemia in NHANES excluding the participants with hypertension (*N* = 2557).

**Table S7:** Associations between blood metal levels and triglycerides in NHANES with additional adjustment for blood Cd, Hg, Pb, Se and Mn levels (*N* = 4182).

**Table S8:** Associations between blood metal levels and hypertriglyceridemia in NHANES with additional adjustment for blood Cd, Hg, Pb, Se and Mn levels (*N* = 4182).

**Table S9:** Associations between blood metal levels and triglycerides in NHANES with additional adjustment for dietary iron, dietary magnesium, and drinking water intake (*N* = 4182).

**Table S10:** Associations between blood metal levels and hypertriglyceridemia in NHANES with additional adjustment for dietary iron, dietary magnesium, and drinking water intake (*N* = 4182).

**Table S11:** Associations between blood metal levels and triglycerides in NHANES with additional adjustment for AST, ALT, HDL, and LDL (*N* = 4182).

**Table S12:** Associations between blood metal levels and hypertriglyceridemia in NHANES with additional adjustment for AST, ALT, HDL, and LDL (*N* = 4182).

## Data Availability

Publicly available datasets were analyzed in this study. These data can be downloaded from: https://www.cdc.gov/nchs/nhanes/.
