# Supplementary figures and images for: Associations Between Blood Metal Exposure and Hypertriglyceridemia Among Adults in NHANES, 2011–2018
Source: Food Sci Nutr. 2025 Sep 21;13(9):e71001. doi: 10.1002/fsn3.71001 (PMC12450778; doi:10.1002/fsn3.71001)

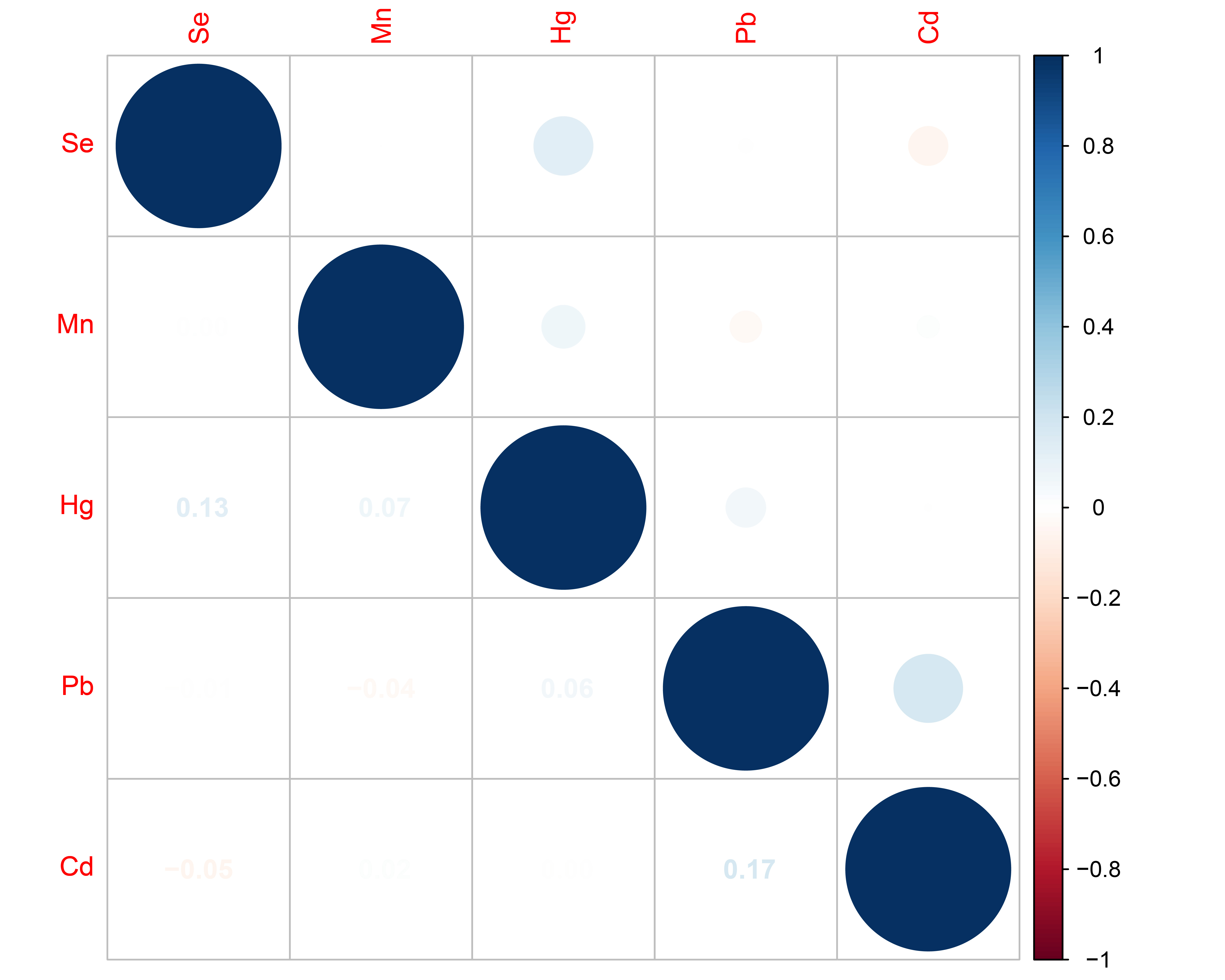


**Fig. S1.** The Pearson correlation between blood metals after In-transformed.

Supplement: Supplementary file 1 — Figure S1: The Pearson correlation between blood metals after In‐transformed. [file FSN3-13-e71001-s013.docx]
