# Supplementary material for: Associations Between Blood Metal Exposure and Hypertriglyceridemia Among Adults in NHANES, 2011–2018
Source: Food Sci Nutr. 2025 Sep 21;13(9):e71001. doi: 10.1002/fsn3.71001 (PMC12450778; doi:10.1002/fsn3.71001)
Supplement: Supplementary file 3 — Figure S3: Estimated weights of blood metals for hypertriglyceridemia in total population (A) and subgroups stratified by age (B‐C) and gender (D‐E) by WQS model. Model was adjusted for gender, age, race/ethnicity, FIPR, educational level, smoking status, drinking alcohol status, BMI, physical activity, total energy intake, HEI‐2015, CKD, diabetes, and hypertension. [file FSN3-13-e71001-s009.docx]

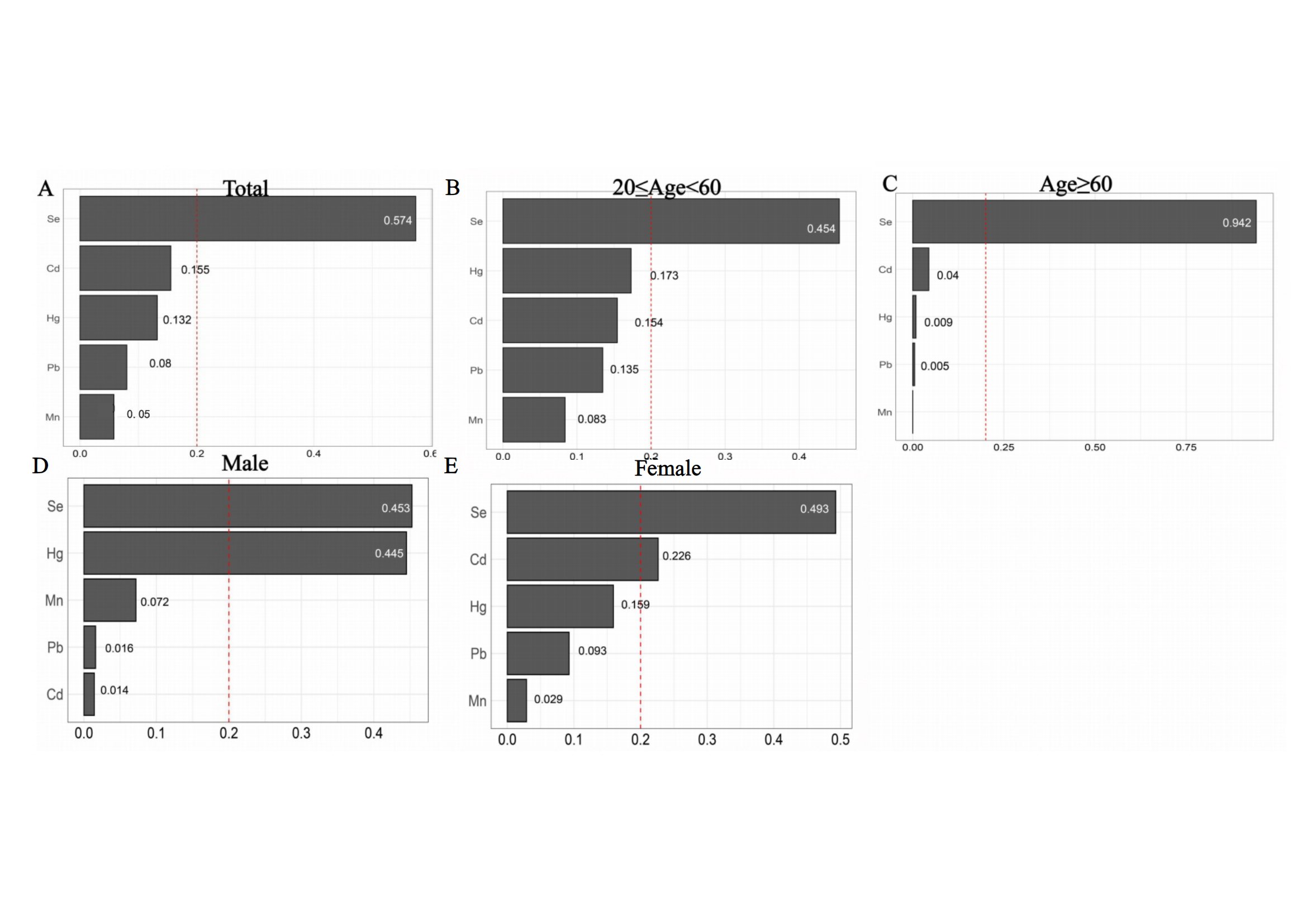


**Fig. S3.** Estimated weights of blood metals for hypertriglyceridemia in total population (A) and subgroups stratified by age (B-C) and gender (D-E) by WQS model. Model was adjusted for gender, age, race/ethnicity, FIPR, educational level, smoking status, drinking alcohol status, BMI, physical activity, total energy intake, HEI-2015, CKD, diabetes, and hypertension.
