# Supplementary material for: Associations Between Blood Metal Exposure and Hypertriglyceridemia Among Adults in NHANES, 2011–2018
Source: Food Sci Nutr. 2025 Sep 21;13(9):e71001. doi: 10.1002/fsn3.71001 (PMC12450778; doi:10.1002/fsn3.71001)
Supplement: Supplementary file 9 — Figure S9: The associations between blood metal levels and hypertriglyceridemia stratified by gender. Model was adjusted for age, race/ethnicity, FIPR, educational level, smoking status, drinking alcohol status, BMI, physical activity, total energy intake, HEI‐2015, CKD, diabetes, and hypertension. [file FSN3-13-e71001-s017.docx]

**Fig. S9.** The associations between blood metal levels and hypertriglyceridemia stratified by gender. Model was adjusted for age, race/ethnicity, FIPR, educational level, smoking status, drinking alcohol status, BMI, physical activity, total energy intake, HEI-2015, CKD, diabetes, and hypertension.
