# Supplementary material for: Associations Between Blood Metal Exposure and Hypertriglyceridemia Among Adults in NHANES, 2011–2018
Source: Food Sci Nutr. 2025 Sep 21;13(9):e71001. doi: 10.1002/fsn3.71001 (PMC12450778; doi:10.1002/fsn3.71001)
Supplement: Supplementary file 11 — Table S1: The distributions of blood metals in the NHANES 2011–2018. [file FSN3-13-e71001-s018.docx]

**Table S1.** The distributions of blood metals in the NHANES 2011-2018.

| Metal (ng/mL) | Detection rates (%) | Mean | GM | P5 | P25 | P50 | P75 | P95 |
| --- | --- | --- | --- | --- | --- | --- | --- | --- |
| Pb (ng/mL) | 100% | 13.46 | 10.22 | 3.4 | 6.3 | 10.1 | 16 | 31.79 |
| Cd (ng/mL) | 99.96% | 0.49 | 0.33 | 0.11 | 0.19 | 0.31 | 0.55 | 1.56 |
| Hg (ng/mL) | 99.77% | 1.4 | 0.82 | 0.2 | 0.4 | 0.76 | 1.52 | 4.68 |
| Se (ng/mL) | 86.82% | 194.05 | 192.34 | 157.5415 | 177.57 | 191.84 | 207.82 | 236.769 |
| Mn (ng/mL) | 100% | 9.99 | 9.4 | 5.38 | 7.52 | 9.35 | 11.67 | 16.519 |
