# Supplementary material for: Associations Between Blood Metal Exposure and Hypertriglyceridemia Among Adults in NHANES, 2011–2018
Source: Food Sci Nutr. 2025 Sep 21;13(9):e71001. doi: 10.1002/fsn3.71001 (PMC12450778; doi:10.1002/fsn3.71001)
Supplement: Supplementary file 12 — Table S2: Determination of variance inflation factor of the variables. [file FSN3-13-e71001-s007.docx]

**Table S2.** Determination of variance inflation factor of the variables.

| **Variable** | **Variance inflation factor** |
| --- | --- |
| Age | 2.119 |
| Gender | 1.366 |
| Race/ethnicity | 1.495 |
| Educational level | 1.409 |
| Marital status | 1.404 |
| FIPR | 1.372 |
| BMI | 1.185 |
| Smoking status | 1.379 |
| Drinking alcohol status | 1.155 |
| Total energy intake | 1.200 |
| Physical activity | 1.317 |
| CKD | 1.210 |
| Diabetes | 1.254 |
| Hypertension | 1.486 |
| HEI-2015 | 1.254 |
