# Supplementary material for: Associations Between Blood Metal Exposure and Hypertriglyceridemia Among Adults in NHANES, 2011–2018
Source: Food Sci Nutr. 2025 Sep 21;13(9):e71001. doi: 10.1002/fsn3.71001 (PMC12450778; doi:10.1002/fsn3.71001)
Supplement: Supplementary file 13 — Table S3: Associations between blood metal levels and triglycerides in NHANES excluding the participants with diabetes (N = 3378). [file FSN3-13-e71001-s003.docx]

**Table S3.** Associations between blood metal levels and triglycerides in NHANES excluding the participants with diabetes (N =3378).

| **Variable** | **Triglycerides β (95% CI)** | | | | | | | |
| --- | --- | --- | --- | --- | --- | --- | --- | --- |
|  | **Categorical variable** | | | | | **Continuous variable** | | |
|  | **T1** | **T2** | **T3** | ***p*-trend** | **Ln-transformed** | | ***p*-value** |  |
| Pb | Reference | -0.01(-0.09, 0.06) | -0.01(-0.10, 0.08) | >0.9 | 0.04(-0.02, 0.10) | | 0.15 |  |
| Cd | Reference | 0.02(-0.06, 0.11) | 0.07(-0.03, 0.17) | 0.4 | 0.05(0.01, 0.09) | | 0.024 |  |
| Hg | Reference | 0.02(-0.07, 0.11) | -0.01(-0.08, 0.07) | 0.8 | -0.01(-0.04, 0.02) | | 0.6 |  |
| Se | Reference | 0.08(-0.01, 0.17) | 0.21(0.12, 0.30) | <0.001 | 0.55(0.22, 0.89) | | <0.001 |  |
| Mn | Reference | 0.04(-0.03, 0.11) | -0.04(-0.11, 0.03) | 0.089 | -0.03(-0.11, 0.06) | | 0.6 |  |

Model was adjusted for gender, age, race/ethnicity, FIPR, educational level, smoking status, drinking alcohol status, BMI, physical activity, total energy intake, HEI-2015, CKD, diabetes, and hypertension.
