# Supplementary material for: Associations Between Blood Metal Exposure and Hypertriglyceridemia Among Adults in NHANES, 2011–2018
Source: Food Sci Nutr. 2025 Sep 21;13(9):e71001. doi: 10.1002/fsn3.71001 (PMC12450778; doi:10.1002/fsn3.71001)
Supplement: Supplementary file 14 — Table S4: Associations between blood metal levels and hypertriglyceridemia in NHANES excluding the participants with diabetes (N = 3378). [file FSN3-13-e71001-s010.docx]

**Table S4.** Associations between blood metal levels and hypertriglyceridemia in NHANES excluding the participants with diabetes (N =3378).

| **Variable** | **Hypertriglyceridemia OR (95% CI)** | | | | | | | |
| --- | --- | --- | --- | --- | --- | --- | --- | --- |
|  | **Categorical variable** | | | | | **Continuous variable** | | |
|  | **T1** | **T2** | **T3** | ***p*-trend** | **Ln-transformed** | | ***p*-value** |  |
| Pb | Reference | 0.87(0.62, 1.21) | 0.83(0.55, 1.26) | 0.6 | 1.09(0.87, 1.36) | | 0.4 |  |
| Cd | Reference | 1.21(0.84, 1.73) | 1.31(0.92, 1.86) | 0.3 | 1.15(0.98, 1.36) | | 0.083 |  |
| Hg | Reference | 1.25(0.94, 1.67) | 1.13(0.82, 1.55) | 0.3 | 1.06(0.93, 1.22) | | 0.3 |  |
| Se | Reference | 1.16(0.82, 1.66) | 1.79(1.28, 2.52) | <0.001 | 4.51(1.60, 12.7) | | 0.003 |  |
| Mn | Reference | 1.22(0.93, 1.61) | 0.88(0.68, 1.14) | 0.055 | 0.93(0.66, 1.29) | | 0.6 |  |

Model was adjusted for gender, age, race/ethnicity, FIPR, educational level, smoking status, drinking alcohol status, BMI, physical activity, total energy intake, HEI-2015, CKD, diabetes, and hypertension.
