# Supplementary material for: Associations Between Blood Metal Exposure and Hypertriglyceridemia Among Adults in NHANES, 2011–2018
Source: Food Sci Nutr. 2025 Sep 21;13(9):e71001. doi: 10.1002/fsn3.71001 (PMC12450778; doi:10.1002/fsn3.71001)
Supplement: Supplementary file 16 — Table S6: Associations between blood metal levels and hypertriglyceridemia in NHANES excluding the participants with hypertension (N = 2557). [file FSN3-13-e71001-s019.docx]

**Table S6.** Associations between blood metal levels and hypertriglyceridemia in NHANES excluding the participants with hypertension (N =2557).

| **Variable** | **Hypertriglyceridemia OR (95% CI)** | | | | | | | |
| --- | --- | --- | --- | --- | --- | --- | --- | --- |
|  | **Categorical variable** | | | | | **Continuous variable** | | |
|  | **T1** | **T2** | **T3** | ***p*-trend** | **Ln-transformed** | | ***p*-value** |  |
| Pb | Reference | 0.9(0.64, 1.25) | 0.98(0.66, 1.44) | 0.8 | 1.03(0.83, 1.28) | | 0.8 |  |
| Cd | Reference | 1.02(0.71, 1.48) | 1.15(0.79, 1.67) | 0.7 | 1.11(0.92, 1.34) | | 0.3 |  |
| Hg | Reference | 1.03(0.71, 1.50) | 0.99(0.69, 1.41) | >0.9 | 1(0.86, 1.16) | | >0.9 |  |
| Se | Reference | 1.18(0.81, 1.70) | 1.72(1.21, 2.44) | 0.003 | 3.21(1.11, 9.27) | | 0.026 |  |
| Mn | Reference | 1.08(0.76, 1.55) | 0.97(0.66, 1.4) | 0.7 | 1.02(0.65, 1.60) | | >0.9 |  |

Model was adjusted for gender, age, race/ethnicity, FIPR, educational level, smoking status, drinking alcohol status, BMI, physical activity, total energy intake, HEI-2015, CKD, diabetes, and hypertension.
