# Supplementary material for: Associations Between Blood Metal Exposure and Hypertriglyceridemia Among Adults in NHANES, 2011–2018
Source: Food Sci Nutr. 2025 Sep 21;13(9):e71001. doi: 10.1002/fsn3.71001 (PMC12450778; doi:10.1002/fsn3.71001)
Supplement: Supplementary file 17 — Table S7: Associations between blood metal levels and triglycerides in NHANES with additional adjustment for blood Cd, Hg, Pb, Se and Mn levels (N = 4182). [file FSN3-13-e71001-s014.docx]

**Table S7.** Associations between blood metal levels and triglycerides in NHANES with additional adjustment for blood Cd, Hg, Pb, Se and Mn levels (N = 4182).

| **Variable** | **Triglycerides β (95% CI)** | | | | | | | |
| --- | --- | --- | --- | --- | --- | --- | --- | --- |
|  | **Categorical variable** | | | | | **Continuous variable** | | |
|  | **T1** | **T2** | **T3** | ***p*-trend** | **Ln-transformed** | | ***p*-value** |  |
| Pb | Reference | 0(-0.08, 0.07) | -0.02(-0.10, 0.07) | >0.9 | 0.03(-0.03, 0.08) | | 0.4 |  |
| Cd | Reference | 0.03(-0.05, 0.11) | 0.08(-0.02, 0.18) | 0.2 | 0.05(0.00, 0.10) | | 0.06 |  |
| Hg | Reference | 0.04(-0.04, 0.13) | 0.01(-0.06, 0.08) | 0.5 | -0.01(-0.04, 0.02) | | 0.5 |  |
| Se | Reference | 0.08(0.00, 0.16) | 0.21(0.14, 0.29) | <0.001 | 0.56(0.25, 0.87) | | <0.001 |  |
| Mn | Reference | 0.01(-0.05, 0.07) | -0.08(-0.14, -0.02) | 0.01 | -0.06(-0.13, 0.01) | | 0.11 |  |

Model was adjusted for gender, age, race/ethnicity, FIPR, educational level, smoking status, drinking alcohol status, BMI, physical activity, total energy intake, HEI-2015, CKD, diabetes, hypertension, blood Cd, blood Hg, blood Pb, blood Se, and blood Mn. Analyses were conducted without adjusting for the metal itself.
