# Supplementary material for: Associations Between Blood Metal Exposure and Hypertriglyceridemia Among Adults in NHANES, 2011–2018
Source: Food Sci Nutr. 2025 Sep 21;13(9):e71001. doi: 10.1002/fsn3.71001 (PMC12450778; doi:10.1002/fsn3.71001)
Supplement: Supplementary file 18 — Table S8: Associations between blood metal levels and hypertriglyceridemia in NHANES with additional adjustment for blood Cd, Hg, Pb, Se and Mn levels (N = 4182). [file FSN3-13-e71001-s004.docx]

**Table S8.** Associations between blood metal levels and hypertriglyceridemia in NHANES with additional adjustment for blood Cd, Hg, Pb Se and Mn levels (N = 4182).

| **Variable** | **Hypertriglyceridemia OR (95% CI)** | | | | | | | |
| --- | --- | --- | --- | --- | --- | --- | --- | --- |
|  | **Categorical variable** | | | | | **Continuous variable** | | |
|  | **T1** | **T2** | **T3** | ***p*-trend** | **Ln-transformed** | | ***p*-value** |  |
| Pb | Reference | 0.85(0.62, 1.18) | 0.78(0.54, 1.11) | 0.4 | 1.02(0.82, 1.28) | | 0.8 |  |
| Cd | Reference | 1.28(0.93, 1.75) | 1.43(1.02, 2.01) | 0.088 | 1.14(0.96, 1.36) | | 0.11 |  |
| Hg | Reference | 1.3(1.00, 1.69) | 1.13(0.86, 1.49) | 0.11 | 1.04(0.91, 1.17) | | 0.6 |  |
| Se | Reference | 1.17(0.88, 1.55) | 1.82(1.39, 2.37) | <0.001 | 4.12(1.66, 10.2) | | 0.002 |  |
| Mn | Reference | 1.08(0.85, 1.37) | 0.73(0.58, 0.91) | 0.005 | 0.8(0.60, 1.06) | | 0.1 |  |

Model was adjusted for gender, age, race/ethnicity, FIPR, educational level, smoking status, drinking alcohol status, BMI, physical activity, total energy intake, HEI-2015, CKD, diabetes, hypertension, blood Cd, blood Hg, blood Pb, blood Se, and blood Mn. Analyses were conducted without adjusting for the metal itself.
