# Supplementary material for: Associations Between Blood Metal Exposure and Hypertriglyceridemia Among Adults in NHANES, 2011–2018
Source: Food Sci Nutr. 2025 Sep 21;13(9):e71001. doi: 10.1002/fsn3.71001 (PMC12450778; doi:10.1002/fsn3.71001)
Supplement: Supplementary file 20 — Table S10: Associations between blood metal levels and hypertriglyceridemia in NHANES with additional adjustment for dietary iron, dietary magnesium, and drinking water intake (N = 4182). [file FSN3-13-e71001-s016.docx]

**Table S10.** Associations between blood metal levels and hypertriglyceridemia in NHANES with additional adjustment for dietary iron, dietary magnesium, and drinking water intake (N = 4182).

| **Variable** | **Hypertriglyceridemia OR (95% CI)** | | | | | | | |
| --- | --- | --- | --- | --- | --- | --- | --- | --- |
|  | **Categorical variable** | | | | | **Continuous variable** | | |
|  | **T1** | **T2** | **T3** | ***p*-trend** | **Ln-transformed** | | ***p*-value** |  |
| Pb | Reference | 0.91(0.67, 1.23) | 0.85(0.60, 1.18) | 0.6 | 1.07(0.87, 1.32) | | 0.5 |  |
| Cd | Reference | 1.22(0.90, 1.64) | 1.28(0.93, 1.75) | 0.2 | 1.13(0.96, 1.33) | | 0.12 |  |
| Hg | Reference | 1.27(0.97, 1.65) | 1.11(0.84, 1.46) | 0.2 | 1.05(0.93, 1.19) | | 0.4 |  |
| Se | Reference | 1.19(0.89, 1.58) | 1.78(1.36, 2.32) | <0.001 | 4.17(1.76, 9.89) | | <0.001 |  |
| Mn | Reference | 1.11(0.87, 1.41) | 0.77(0.62, 0.95) | 0.013 | 0.84(0.64, 1.10) | | 0.2 |  |

Model was adjusted for gender, age, race/ethnicity, FIPR, educational level, smoking status, drinking alcohol status, BMI, physical activity, total energy intake, HEI-2015, CKD, diabetes, hypertension, dietary iron, dietary magnesium, and drinking water intake.
