# Supplementary material for: Associations Between Blood Metal Exposure and Hypertriglyceridemia Among Adults in NHANES, 2011–2018
Source: Food Sci Nutr. 2025 Sep 21;13(9):e71001. doi: 10.1002/fsn3.71001 (PMC12450778; doi:10.1002/fsn3.71001)
Supplement: Supplementary file 21 — Table S11: Associations between blood metal levels and triglycerides in NHANES with additional adjustment for AST, ALT, HDL, and LDL (N = 4182). [file FSN3-13-e71001-s021.docx]

**Table S11.** Associations between blood metal levels and triglycerides in NHANES with additional adjustment for AST, ALT, HDL, and LDL (N =4182).

| **Variable** | **Triglycerides β (95% CI)** | | | | | | | |
| --- | --- | --- | --- | --- | --- | --- | --- | --- |
|  | **Categorical variable** | | | | | **Continuous variable** | | |
|  | **T1** | **T2** | **T3** | ***p*-trend** | **Ln-transformed** | | ***p*-value** |  |
| Pb | Reference | 0(-0.07, 0.07) | 0.01(-0.07, 0.08) | >0.9 | 0.04(-0.01, 0.09) | | 0.15 |  |
| Cd | Reference | 0.01(-0.06, 0.08) | 0.05(-0.03, 0.14) | 0.3 | 0.03(-0.01, 0.08) | | 0.12 |  |
| Hg | Reference | 0.04(-0.04, 0.12) | 0.02(-0.05, 0.09) | 0.6 | 0(-0.03, 0.03) | | >0.9 |  |
| Se | Reference | 0.06(-0.01, 0.14) | 0.16(0.09, 0.24) | <0.001 | 0.38(0.10, 0.65) | | 0.006 |  |
| Mn | Reference | -0.01(-0.07, 0.06) | -0.08(-0.13, -0.02) | 0.008 | -0.06(-0.13, 0.01) | | 0.065 |  |

Model was adjusted for gender, age, race/ethnicity, FIPR, educational level, smoking status, drinking alcohol status, BMI, physical activity, total energy intake, HEI-2015, CKD, diabetes, hypertension, AST, ALT, HDL, and LDL.
