# Supplementary material for: Associations Between Blood Metal Exposure and Hypertriglyceridemia Among Adults in NHANES, 2011–2018
Source: Food Sci Nutr. 2025 Sep 21;13(9):e71001. doi: 10.1002/fsn3.71001 (PMC12450778; doi:10.1002/fsn3.71001)
Supplement: Supplementary file 22 — Table S12: Associations between blood metal levels and hypertriglyceridemia in NHANES with additional adjustment for AST, ALT, HDL, and LDL (N = 4182). [file FSN3-13-e71001-s012.docx]

**Table S12.** Associations between blood metal levels and hypertriglyceridemia in NHANES with additional adjustment for AST, ALT, HDL, and LDL (N =4182).

| **Variable** | **Hypertriglyceridemia OR (95% CI)** | | | | | | | |
| --- | --- | --- | --- | --- | --- | --- | --- | --- |
|  | **Categorical variable** | | | | | **Continuous variable** | | |
|  | **T1** | **T2** | **T3** | ***p*-trend** | **Ln-transformed** | | ***p*-value** |  |
| Pb | Reference | 0.86(0.62, 1.19) | 0.83(0.58, 1.19) | 0.5 | 1.07(0.87, 1.32) | | 0.5 |  |
| Cd | Reference | 1.3(0.95, 1.77) | 1.32(0.91, 1.9) | 0.2 | 1.11(0.92, 1.33) | | 0.3 |  |
| Hg | Reference | 1.33(1.01, 1.75) | 1.16(0.82, 1.64) | 0.09 | 1.07(0.92, 1.25) | | 0.3 |  |
| Se | Reference | 1.08(0.79, 1.48) | 1.52(1.15, 2.01) | 0.003 | 2.51(1.02, 6.15) | | 0.038 |  |
| Mn | Reference | 0.99(0.74, 1.31) | 0.7(0.55, 0.89) | 0.005 | 0.71(0.53, 0.95) | | 0.017 |  |

Model was adjusted for gender, age, race/ethnicity, FIPR, educational level, smoking status, drinking alcohol status, BMI, physical activity, total energy intake, HEI-2015, CKD, diabetes, hypertension, AST, ALT, HDL, and LDL.
